# Codon Usage Bias and Phylogenetic Analysis of the Mitochondrial Genomes in Two *Enicurus* Species

**DOI:** 10.3390/genes17050518

**Published:** 2026-04-28

**Authors:** Lifu Qian, Jiahao Zan, Han Liu, Tao Liu, Jinming Zhao, Xiaoming Li

**Affiliations:** Anhui Engineering Research Center for Green Production Technology of Drought Grain Crops, College of Life Sciences, Huaibei Normal University, Huaibei 235000, China; zjh15551988819@163.com (J.Z.); 19577384421@163.com (H.L.); liut202604@163.com (T.L.); zhaojm2022@163.com (J.Z.)

**Keywords:** *Enicurus*, mitochondrial genome, codon usage bias, natural selection, optimal codon, phylogenetic analysis

## Abstract

**Background:** Codon usage bias (CUB), which is shaped by mutation pressure, natural selection, and genetic drift, provides valuable insights into phylogenetic relationships and molecular evolution. This study investigated the patterns and determinants of mitochondrial genome codon usage in two *Enicurus* species (*Enicurus scouleri* and *Enicurus schistaceus*) and provided a foundation for understanding codon optimisation mechanisms and genetic relationships within this avian genus. **Methods:** Complete mitochondrial genome sequences were retrieved from GenBank, and ten protein-coding sequences were selected for CUB analysis. Evolutionary relationships across the studied species were investigated using phylogenetic trees and relative synonymous codon usage (RSCU) clustering diagrams. **Results:** GC1, GC2, and GC3 contents were below 50% in both species, with the third-position nucleotides exhibiting A3s > C3s > T3s > G3s composition. The average effective number of codons (ENC) value was >35, indicating a weak bias for codon usage. CUB reflects the combined effects of natural selection and mutational pressure, with the former exerting a stronger influence. Four shared optimal codons were identified with a strong bias towards A/C-ending triplets. Subsequent phylogenetic analysis validated the close kinship of the two *Enicurus* species, although RSCU-based clustering yielded results that diverged from the phylogenetic relationships. **Conclusions:** Comprehensive mechanistic analysis revealed natural selection as the dominant force shaping mitochondrial CUB in *Enicurus* species. The findings offered valuable insights for future research on the reproductive biology, environmental adaptation, and conservation of *Enicurus* birds while providing new perspectives on the molecular evolution and systematic development of Muscicapidae.

## 1. Introduction

Codons are crucial structural units that convey genetic information from nucleic acids to proteins. Genetic information stored in nucleic acids is transferred to proteins via triplet codons; each amino acid is encoded by a minimum of one codon and a maximum of six codons. Except for methionine and tryptophan, synonymous codons can encode the same amino acid, indicating the degenerative nature of the genetic code [1]. Codon usage bias (CUB) describes the non-random occurrence of synonymous codons in genes, most evident in the third position of codons [2]. Species commonly prefer to use specific codons, which form a unique codon usage pattern for that species and are similar across all species within the same genus. For a species or in a genome, at least one specific synonymous codon appears frequently and is referred to as an optimal codon; in contrast, some codons, named rare codons, are rarely used or may not appear at all. In the genome, different genes exhibit different codon biases, and mutations pressure and natural selection are the two key factors shaping the CUB in different species [3,4,5,6]. Research has shown that CUB among species is influenced by various factors, such as gene expression levels, gene length, base composition, tRNA abundance, GC content, environmental pressure, and population size [6,7,8,9,10,11,12,13]. CUB not only captures the evolutionary origins, mutation patterns, evolutionary trajectories of species or genes, and the phylogenetic relationships among species, but also holds significant importance for studying natural selection, genetic drift, and genetic recombination, thereby helping the elucidation of molecular mechanisms underlying the adaptation of species to the environment [2,6,14,15,16,17,18].

Mitochondria is a key organelle responsible for essential cellular processes, such as metabolism, respiration, and energy conversion. As a semi-autonomous organelle, it has an independent genetic system that is capable of autonomous replication, transcription, and translation, with some exhibiting unique characteristics, such as maternal inheritance, small size, high evolutionary conservation, and the absence of introns [19]. The mitochondrial genome encodes proteins involved in metabolic processes, such as respiration, and contains significant genetic information. The continuous evolution of molecular biotechnology and growing availability of whole-genome sequence data have led to a surge in CUB-related studies across the mitochondrial genomes of diverse species. At present, mitochondrial genome codon usage analyses have been conducted extensively across diverse aquatic taxa, including *Melo melo*, *Pinctada margaritifera*, and *Sipunculus nudus*, as well as insect species, such as *Japanagallia*, *Arma custos*, *Xylotrechus quadripes*, and *Bombyx mori* [14,20,21,22,23,24,25,26]. Furthermore, systematic investigations of mitochondrial CUB have been reported across diverse vertebrate groups, with particular focus on studying the evolutionary constraints (natural selection and mutation pressure) of synonymous codon optimisation in the respiratory chain and oxidative phosphorylation genes. Meng et al. studied codon usage in the mitochondrial genomes of *Oplegnathus spunctatus* and *Oplegnathus fasciatus* and found that the genes encoding proteins preferentially used codons ending in AC, with UUC and ACC being the preferred ones [27]. Previous studies had investigated codon usage patterns in amphibian mitochondrial genomes. For instance, Jiang et al. performed a bioinformatics-based clustering analysis of Hynobiidae species, revealing that natural selection predominates over mutational pressure in shaping the codon preferences of mitochondrial protein-coding genes [28]. In a broad-scale analysis of 22 Certhioidea mitogenomes, Ding et al. demonstrated that CUB exhibits gene-specific asymmetry, with natural selection emerging as the predominant driver of codon usage evolution [29]. Analyses of CUB clustering and the effective number of codons (ENC) in 400 reptile mitochondrial genomes revealed compositional asymmetry based on relative synonymous codon usage (RSCU) and indicated that reptile mitochondrial genomes exhibit high translational efficiency and are subject to selection pressure [30]. Although research on CUB in animal mitochondrial genomes has made continuous progress, no systematic studies on codon usage in *Enicurus* species have been reported. Moreover, the factors contributing to this bias and the relationship between the bias and *Enicurus* evolution remain unclear.

The forktail *Enicurus* (Temminck, 1822), which belongs to the family Muscicapidae, has 11 subspecies from eight species [31]. All species of the genus *Enicurus* have striking black and white streaked feathers, elongated and deeply forked tail feathers, and simple sharp calls [32]. At present, the eight forktail species are mainly distributed in the mountain stream habitats of the Himalaya–Hengduan Mountain region in China (including the Central Mountain Range of Taiwan) and the Indochinese Peninsula to the Indonesian archipelago, and they capture invertebrates from water edges, leaf litter, and flowing water surfaces [33,34,35]. Current research primarily focuses on the phylogeny, biogeography, and breeding biology of *Enicurus* [34,36,37,38,39,40,41,42].

Ever since the first chicken mitogenome was reported, continuous advancement of sequencing technologies has led to a rapid increase in the number of avian mitogenomes. However, within the genus *Enicurus*, only two complete mitogenomes and a few sequenced mitochondrial genes (such as *ND2*, *ND3*, *COX1*, *ATP6*, and *CYTB*) exist, which have severely constrained the investigation of phylogenetic relationships within the taxon and remain insufficient to support comprehensive phylogenetic analyses of the family Muscicapidae. However, studies on the mitochondrial genome of *Enicurus* remain limited, particularly in terms of comprehensive analyses of CUB. In this study, we aimed to compare the CUB of mitochondrial core protein-coding genes between *Enicurus scouleri* and *Enicurus schistaceus*. We explored the codon composition characteristics and factors of codon usage preference, and identified the optimal codons. Furthermore, a phylogenetic tree of the family Muscicapidae was constructed using the RSCU values and coding sequences of the mitochondrial genome codons. The results derived from this study could possibly help understand codon usage and molecular evolution in the mitochondrial genome of *Enicurus*, and provide a reference for in-depth research on mitogenome gene utilisation and phylogenetic evolutionary relationships of Muscicapidae birds.

## 2. Materials and Methods

### 2.1. Sequence Retrieval

The complete mitochondrial genome sequences of *E. scouleri* (accession number OP998296) and *E. schistaceus* (accession number PP663688) were deposited in the NCBI database (https://www.ncbi.nlm.nih.gov). In this study, we downloaded 13 core protein-coding genes of the two *Enicurus* mitogenomes from the NCBI database to analyse codon usage. To ensure data quality, core protein-coding genes were filtered to exclude sequences < 300 bp in length, non-ATG initiators, and repetitive regions [43]. This yielded the following ten core protein-coding genes per *Enicurus* mitogenome for downstream analysis: *ND1*, *ND2*, *ND3*, *ND4*, *ND5*, *ND6*, *ATP6*, *COX2*, *COX3*, and *CYTB*. To broaden the phylogenetic context, core protein-coding genes from 28 Muscicapidae species were obtained from the NCBI database and combined with the data from the two *Enicurus* species for phylogenetic and cluster analyses (Table S1).

### 2.2. Analysis of Codon Usage Characteristic Indices

Codon usage indicators of the 10 core protein-coding genes in the two *Enicurus* species were analysed using CodonW v1.4.2. with default parameters, including GC of the silent 3rd codon position (GC3s), frequency of mononucleotides at the third codon position (A3, C3, T3, and G3 percentages), codon adaption index (CAI), codon bias index (CBI), frequency of optimal codon (Fop), ENC, RSCU, number of synonymous codons (L-sym), total number of amino acids (L-aa), grand average of hydropathicity (Gravy), and aromaticity of proteins (Aromo). The meaning of each index as follows: Codon Adaptation Index (CAI): a measurement of the relative adaptiveness of the codon usage of a gene towards the codon usage of highly expressed genes; values close to 1 indicate stronger adaptation. Codon Bias Index (CBI): a measure of directional codon bias, it measures the extent to which a gene uses a subset of optimal codons. Frequency of Optimal codons (Fop): the ratio of optimal codons to synonymous codons (genetic code dependent). ENC-observed or effective number of codons (ENC): a simple measure of overall codon bias and is analogous to the effective number of alleles measure used in population genetics. Relative Synonymous Codon Usage (RSCU): observed frequency of a codon divided by the frequency expected under equal usage. Length of synonymous codons (L_sym): the index represents the number of synonymous codons. Length of amino acids (L_aa): the index represents the number of translatable codons. General average hydropathicity (GRAVY): the index is used for the hypothetical translated gene product, calculated as the arithmetic mean of the sum of the hydropathic indices of each amino acid. Amoro: proportion of aromatic amino acids (Phe, Tyr, Trp) among all amino acids. Other codon composition indices (GCall, GC1, GC2, and GC3) were calculated using the online CUSP program in the EMBOSS suite (https://www.bioinformatics.nl/cgi-bin/emboss/cusp (accessed on 8 May 2025)) with default parameters. Following the calculation of these indices, a correlation analysis of the main characteristic parameters was performed using the Correlation Plot tool in Origin 2024 software.

### 2.3. Multivariate Statistical Analysis of Codon Bias

#### 2.3.1. Neutrality Plot Analysis

Neutrality plots examined the correlations between bases at three codon positions to disentangle the effects of mutation pressure and natural selection on codon bias formation [44]. First, we calculated the average value GC12 based on the codons GC1 and GC2. A scatter plot with GC12 on the *y*-axis and GC3 on the *x*-axis was constructed, where each point represented a gene. Finally, a linear regression line was drawn to show the relationship between GC12 and GC3. The slope of the regression line indicated the dominant factor influencing codon usage preferences. When GC12 and GC3 were significantly correlated, with points falling near the diagonal and a slope approaching 1, mutation pressure was inferred to be the main determinant of codon usage bias. Conversely, if the slope was closer to zero, with no significant correlation between GC12 and GC3, natural selection was the dominant factor influencing codon usage [15,44,45,46].

#### 2.3.2. ENC-Plot Analysis

ENC is used to quantify the extent to which codon usage deviates from random selection [47]. Its value ranges from 20 to 61, and is negatively correlated with codon preference. ENC values are inversely correlated with codon bias strength, as follows: 20 indicates strong preference, >35 indicates weak preference, and 61 indicates no preference [48]. GC3s denotes the average GC content at the third position of synonymous codons and serves as a crucial indicator of deviations in nucleotide composition. ENC plots are frequently employed to investigate the factors influencing genomic codon usage patterns and the interplay between gene base composition and codon bias [47]. To create this plot, a two-dimensional scatter plot was drawn with the ENC value on the vertical axis and GC3s on the horizontal axis. The standard curve for ENC values was included in the plot, using the following formula: ENC_exp_ = 2 + GC3s + 29/((GC3s)^2^ + (1 − GC3s)^2^) [47]. When the scatter points were on or near the standard curve, codon preference was understood to be primarily shaped by mutation pressure. Conversely, when the scatter points deviated significantly from the standard curve, the CUB was considered to be influenced by factors such as natural selection [49]. Distribution of the ENC ratio could assess the discrepancy between the expected ENC values (ENC_exp_) and observed ENC values (ENC_obs_) more effectively. The formula for calculating the ENC ratio is: ENC ratio = (ENC_exp_ − ENC_obs_)/ENC_exp_. When the values of ENC_exp_ and ENC_obs_ were close, the gene codon usage was considered to be predominantly influenced by mutation pressure. Conversely, the remarkable divergence between the values suggested natural selection as the primary force shaping codon usage.

#### 2.3.3. PR2 Plot Analysis

PR2 plot analyses the third-base composition of synonymous codons to assess A/T versus C/G bias, helping to determine whether CUB stems from natural selection, mutation pressure, or other factors. A two-dimensional scatter plot was created using A3s/(A3s + T3s) on the *y*-axis and G3s/(G3s + C3s) on the *x*-axis. The intersection (0.5, 0.5) indicated perfect parity (A = T, G = C). Here, A3s, T3s, G3s and C3s indices were calculated per gene within species. Clustering at this centre suggested a mutation-driven equilibrium, whereas peripheral dispersion indicated directional selection or mutational bias [50]. The equilibrium between A/T and C/G at synonymous sites indicated mutation pressure as the primary force shaping CUB. Conversely, if the points were unevenly distributed around the centre, it would mean that CUB was influenced by both natural selection and other factors.

#### 2.3.4. Correspondence Analysis (COA)

COA is a multivariate statistical method used to analyse RSCU variation and map gene distribution in a multidimensional space [51]. COA generates a series of orthogonal axes that capture trends explaining the variation within a dataset, with each successive axis accounting for progressively less variation. The first axis (Axis 1) accounts for the largest portion of the variation observed in codon usage. In the present study, COA was carried out using CodonW v1.4.2 software (default settings), based on the RSCU values of codons. To identify the determinants of codon bias, Pearson correlation coefficients were computed between the first axis values, and nine key indices (CAI, CBI, Fop, ENC, GC3s, GC1, GC2, GC3, and GC) were assessed using Origin v24.0 and SPSS v25.0.

### 2.4. RSCU and Optimal Codon Identification

The RSCU values reflected the relative preference for synonymous codons (RSCU = 1, no bias; >1, overrepresented; <1, underrepresented). To identify the optimal codons, genes were stratified by ENC into high-expression (top 20%) and low-expression (bottom 20%) groups. Codons with ΔRSCU > 0.08 (RSCU_high − RSCU_low) were classified as high-expression codons. Optimal codons were operationally defined as those exhibiting both ΔRSCU > 0.08 and RSCU > 1 [52].

### 2.5. CUB Clustering and Phylogenetic Analysis

In this study, two approaches were employed to investigate the phylogenetic relationships among Muscicapidae species. The first approach involved a phylogenetic analysis utilizing the protein-coding genes of the mitochondrial genome, while the second consisted of a cluster analysis based on the RSCU values of codons within the same genome. Clustering analysis of RSCU values was conducted using SPSS v25.0 for 59 sense codons (excluding three terminators plus AUG and UGG) across 30 Muscicapidae species (two *Enicurus* and 28 congeners). The analysis employed between-group linkage with the squared Euclidean distance metric to generate a dendrogram [14]. A phylogenetic tree was constructed for 30 species of the family Muscicapidae, employing both Bayesian inference (BI) and maximum likelihood (ML) approaches applied to concatenated protein-coding sequences, with *Sturnus vulgaris* (NC_029360) as the outgroup. Alignment of individual mitochondrial genes was performed using MAFFT v7.4.0, followed by the concatenation of the aligned sequences into a unified dataset [53]. The optimal partitioning scheme and nucleotide substitution models were determined using PartitionFinder v2.1.1 (Table S2) [54].

Bayesian analysis (MrBayes v3.2.6) [55] used random starting trees, four chains (one cold and three heated), and 1,000,000 generations with sampling every 100 generations. Convergence was assessed using the split frequency standard deviation (<0.01) across duplicate runs with 25% burn-in discarded. Posterior probabilities (PP) were calculated using the post-burn-in consensus. Maximum likelihood analysis (RAxML v8.2.12) employed the GTR + G model with 1000 bootstraps [56]. Trees were visualised using Figtree v1.4.3 software.

## 3. Results

### 3.1. Codon Usage Index Analysis

**Figure 1:**
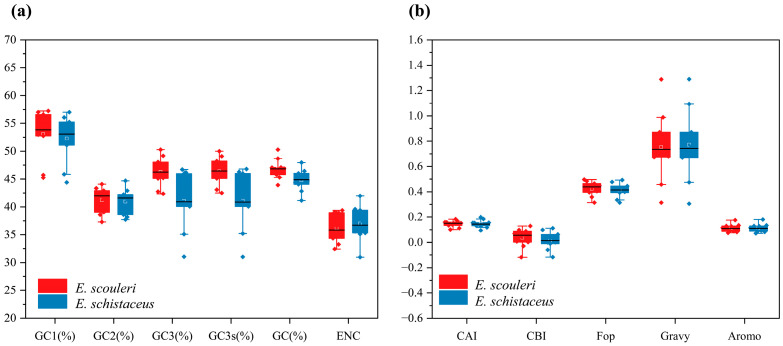
Comprehensive analysis of codon usage in ten mitochondrial genes from two *Enicurus* species. (**a**) Nucleotide composition: total GC (GCall), position-specific GC (GC1–GC3, GC3s), and ENC (Effective Number of Codons, observed). (**b**) Codon adaptation indices: CAI (Codon Adaptation Index), CBI (Codon Bias Index), Fop (Frequency of Optimal Codons), General average hydropathicity (GRAVY), Amoro (proportion of aromatic amino acids). The horizontal line within the box indicates the median of the dataset. The upper and lower boundaries of the box represent the upper quartile (Q3, the 75th percentile) and the lower quartile (Q1, the 25th percentile), respectively.

The GCall content of ten mitochondrial genes in the two *Enicurus* species was 46.63% (*E. scouleri*) and 45.06% (*E. schistaceus*), respectively (Table 1). In both, the GC content at the 1st, 2nd, and 3rd positions of the codons was all less than 50%, differing at each position (Figure 1). Nucleotide compositions at the third synonymous position (A3s, T3s, G3s, and C3s) were calculated, and displayed a trend of A3s > C3s >T3s > G3s, indicating that the 3rd position of codons in the two *Enicurus* species was more inclined to end with A/C (Table 1).

In addition, the CAI, CBI, and FOP values of both *E. scouleri* and *E. schistaceus* were all significantly less than 1 (Figure 1 and Table S3), with CAI concentrated between 0.10–0.18 and 0.10–0.20, respectively. The CBI values were close to 0, and the FOP values were all less than 0.5, suggesting weak CUB and relatively low gene expression levels in ten mitochondrial genes of *Enicurus* species. The ENC values of the two *Enicurus* coding genes ranged from 33.27 to 39.39 (*E. scouleri*) and 30.94 to 41.96 (*E. schistaceus*), respectively. Strong codon bias (ENC < 35) was detected in four *E. scouleri* mitochondrial genes (*ATP6*, *ND3*, *ND5*, and *CYTB*) and one *E. schistaceus* gene (*ND3*); the remaining genes showed weaker bias, with ENC values slightly surpassing 35.

### 3.2. Correlation Analysis Across the CUB Parameters

**Figure 2:**
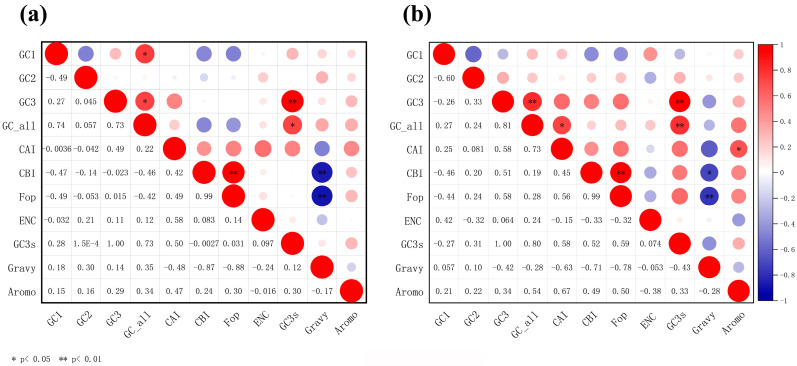
Heatmap visualisation of pairwise correlations among the codon usage parameters for ten mitochondrial genes. (**a**) *E. scouleri*, (**b**) *E. schistaceus*. Colour scale: red (negative) to blue (positive). Size scale: larger circles indicate stronger correlations, smaller circles indicate weaker correlations. ENC stands for ENC observed (Effective Number of Codons, observed). Statistical significance: * *p* < 0.05, ** *p* < 0.01.

The correlation analysis of the main indicators of mitochondrial CUB is shown in Figure 2. For both *Enicurus* species, the FOP exhibited highly significant positive correlations with the CBI (*r* = 0.99, *p* < 0.01 for both). Within the species *E. scouleri* and *E. schistaceus*, GC3 and GC3s were perfectly correlated (*r* = 1.00, *p* < 0.01), while Gravy showed significant negative correlations with FOP (*r* = −0.88 and −0.78, respectively, *p* < 0.01). In *E. scouleri*, GCall was significantly correlated with GC1, GC3, and GC3s (*r* = 0.74, 0.73, and 0.73, respectively, *p* < 0.05), and Gravy was significantly negatively correlated with CBI (*r* = −0.87, *p* < 0.01). For *E. schistaceus*, GCall exhibited a significant positive correlation with GC3 and GC3s (*r* = 0.81 and 0.80, respectively, *p* < 0.01). CAI was positively correlated with GCall and Aromo (*r* = 0.73 and 0.67, *p* < 0.05) while Gravy showed a significant negative correlation with CBI (*r* = −0.71, *p* < 0.05).

### 3.3. Cause Analysis for CUB

#### 3.3.1. Results of Neutrality Plot Analysis

**Figure 3:**
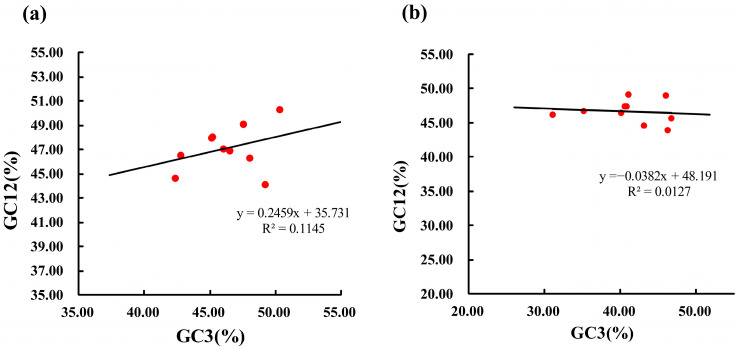
Neutrality plots of ten mitochondrial genes in two *Enicurus* species. (**a**) *E. scouleri*, *ATP6* and *ND4* genes exhibit similar values, with their data points clustering closely together. (**b**) *E. schistaceus*, *COX2* and *ND1* genes exhibit similar values, with their data points clustering closely together.

The GC content at different codon positions was compared between the two *Enicurus* species. Neutrality plots (GC12 vs. GC3) were generated to assess the relationship between the variables and infer the evolutionary forces shaping CUB (Figure 3). For *E. scouleri*, the range of GC3 was 0.4236–0.5029 and that of GC12 was 0.4416–0.5029, with average values of 0.4629 and 0.4712, respectively. For *E. schistaceus*, the range of GC3 was 0.3103–0.4672 and that of GC12 was 0.4389–0.4914, with average values of 0.4106 and 0.4662, respectively. Neutrality analysis revealed no significant GC12-GC3 correlation in either *Enicurus* species (*r* = 0.1145 and 0.0127; *p* > 0.05), with shallow regression slopes (0.2459 and −0.0382) and dispersed data points. The departure from mutational equilibrium (slope near 0) implicated natural selection as the dominant force governing codon preferences.

#### 3.3.2. ENC Plot Analysis

**Figure 4:**
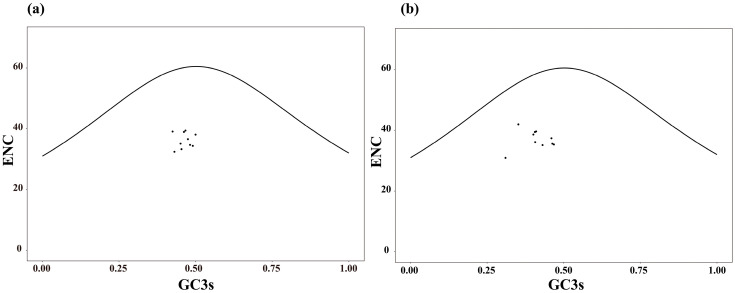
ENC plot analysis of ten mitochondrial genes in two *Enicurus* species. The black line represents the expected curve when the codon usage bias was determined only by mutation pressure. (**a**) *E. scouleri*, (**b**) *E. schistaceus*.

The ENC plot showed that the observed ENC values for both *Enicurus* species consistently fell below the expected standard curve (Figure 4). Such a systematic deviation from mutational equilibrium expectations implied directional natural selection or additional selective constraints as the dominant forces influencing CUB. To enable a more accurate evaluation of ENC value differences, we examined the ENC_ratio_ distribution of the genes under study. The statistical results of ENC_ratio_ frequency showed that for both *Enicurus* species, four genes were distributed in the 0.41–0.45 range, with a frequency of 40%; three and two genes were, respectively, distributed in the 0.36–0.40 range, with frequencies of 30% and 20% (Table 2). Notably, ENC_ratio_ of all genes greater than 0, and exhibited a substantial discrepancy between actual and expected values. These findings further support that natural selection is the primary factor shaping CUB patterns in the mitochondrial genomes of both *Enicurus* species.

#### 3.3.3. Results of PR2 Plot Analysis

**Figure 5:**
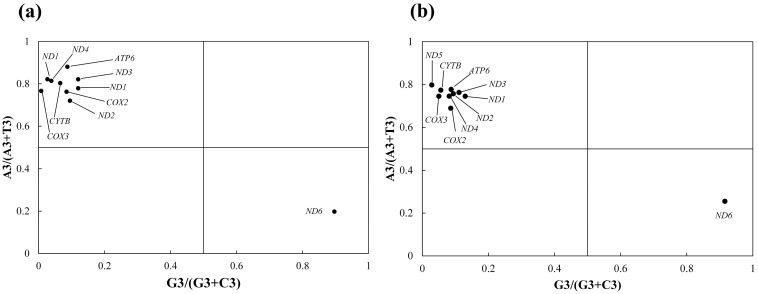
PR2 plots showing third-position nucleotide bias in (**a**) *E. scouleri* and (**b**) *E. schistaceus*. The central point (0.5, 0.5) indicates A = T and G = C equilibrium (no bias).

To further dissect the CUB and elucidate the relative contributions of mutation pressure and natural selection forces, we performed a PR2 plot analysis on the third-position nucleotides of synonymous codons in *Enicurus* mitochondrial genes. The PR2 plots were highly concordant between the two species (Figure 5). Nine genes clustered in the second quadrant (G3/(G3 + C3) < 0.5, A3/(A3 + T3) > 0.5), indicating a predominant preference for A and C at the third position. Only *ND6* deviated to the fourth quadrant, exhibiting a T/G bias. Both species showed higher frequencies of C over G and that of A over T (U) at the third codon position. Notably, all genes in *Enicurus* displayed substantial deviation from the central equilibrium point (0.5, 0.5), indicating a strong CUB shaped by the interplay of natural selection, mutation pressure, and other evolutionary forces.

#### 3.3.4. Correspondence Analysis

**Figure 6:**
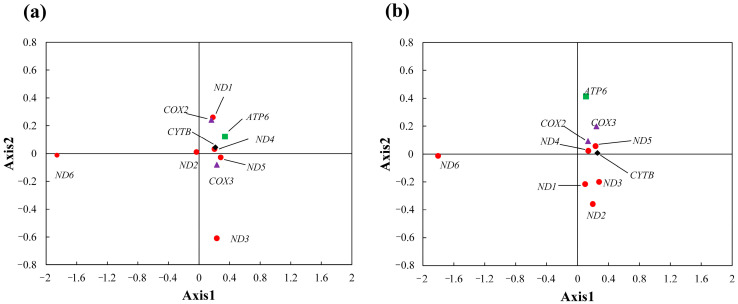
COA of ten mitochondrial genes in *Enicurus* speices. (**a**) *E. scouleri*, (**b**) *E. schistaceus*.

We evaluated the RSCU patterns using correspondence analysis. A coordinate system was constructed with Axis 1 as the *x*-axis and Axis 2 as the *y*-axis (Figure 6). Analysis of the gene distribution on the coordinate axes revealed that in *E. scouleri*, most genes clustered closely while *ND6* and *ND3* were more dispersed. In *E. schistaceus*, *ND1*, *ND2*, and *ND3* were relatively close, whereas *COX2*, *COX3*, *ND4*, *ND5*, and *CYTB* were more concentrated. The other two genes, *ND6* and *ATP6*, were highly dispersed. The distribution patterns indicated that *ND6*, *ND3*, and *ATP6* had significantly different codon biases than other genes.

**Figure 7:**
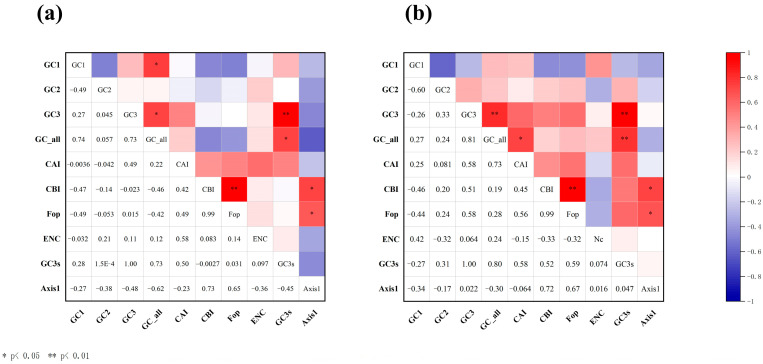
COA correlations between Axis 1 and codon usage indices. Red-to-blue gradient shows increasing correlation. (**a**) *E. scouleri*, (**b**) *E. schistaceus*. Significance: * *p* < 0.05, ** *p* < 0.01.

For the two *Enicurus* species, the first four principal axes accounted for 49.57% and 44.03% of the total variation, respectively, with the first axis alone contributing 38.20% and 34.34% of the variation, respectively. The results showed that the first axis accounted for the greatest proportion of variation, with subsequent axes contributing progressively smaller amounts, indicating that CUB in *Enicurus* mitochondrial genes was shaped by multiple interacting factors. To identify the drivers of gene distribution in the COA plane, we performed correlation analyses between the first axis and GC1, GC2, GC3, GC3s, GCall, CAI, CBI, Fop, and ENC (Figure 7). Pearson correlation analysis revealed significant positive correlations between the first axis and both CBI and FOP values (*E. scouleri*: *r* = 0.73 and 0.65; *E. schistaceus*: *r* = 0.72 and 0.67, respectively), indicating that the translational efficiency indices substantially influence codon usage patterns in both species.

### 3.4. Determination of RSCU Values and Putative Optimal Codons

RSCU analysis revealed that both *Enicurus* species possessed 27 high-frequency codons with RSCU > 1 (Table 3). Notably, all 27 high-frequency codons terminated in either A (13) or C (14) with complete absence of G- and T-ending codons, hence revealing an extreme A/C bias at the third position. Additionally, 24 and 21 high-expression codons (ΔRSCU > 0.08) were identified in *E. scouleri* and *E. schistaceus*, respectively (Table 3). Optimal codons were identified based on dual criteria, namely RSCU > 1 (high-frequency) and ΔRSCU > 0.08 (high-expression), requiring simultaneous satisfaction of both thresholds. A comparative analysis identified 16 and 11 optimal codons in *E. scouleri* and *E. schistaceus*, respectively (Table 4), with four common optimal codons (AUC, GUA, UCC, and CGA) shared between them. The third position of the four optimal codons ended with A/C.

### 3.5. Phylogenetic and Cluster Analysis

To achieve a better understanding of the differences in mitogenome codon usage, cluster analysis based on RSCU was performed between the two *Enicurus* species and their related species. Since *E. scouleri* and *E. schistaceus* are the only two members of the *Enicurus* genus that have undergone complete mitochondrial genome sequencing, an additional 28 Muscicapidae species with available published mitochondrial genome data were selected for further comparative studies.

**Figure 8:**
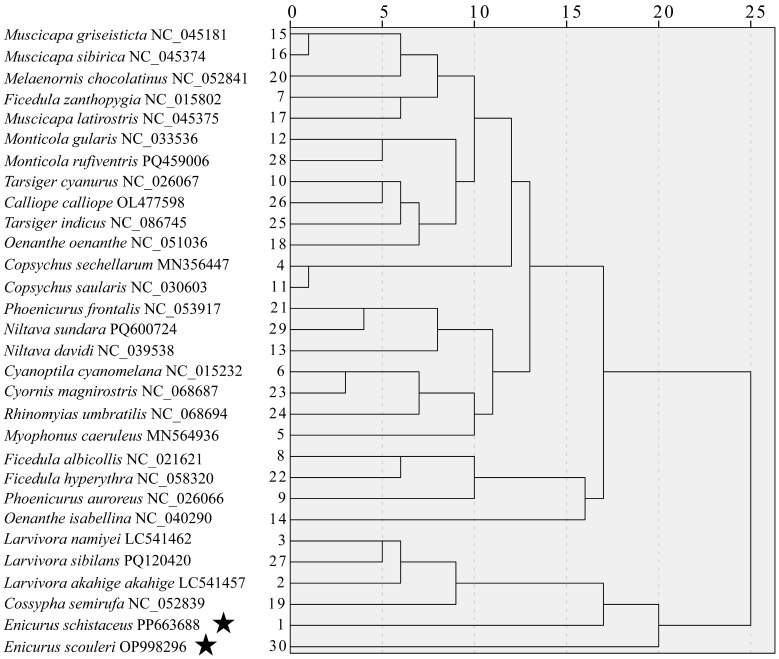
RSCU-based clustering analysis of 30 Muscicapidae. The newly described species in this work are marked with an asterisk next to their names.

**Figure 9:**
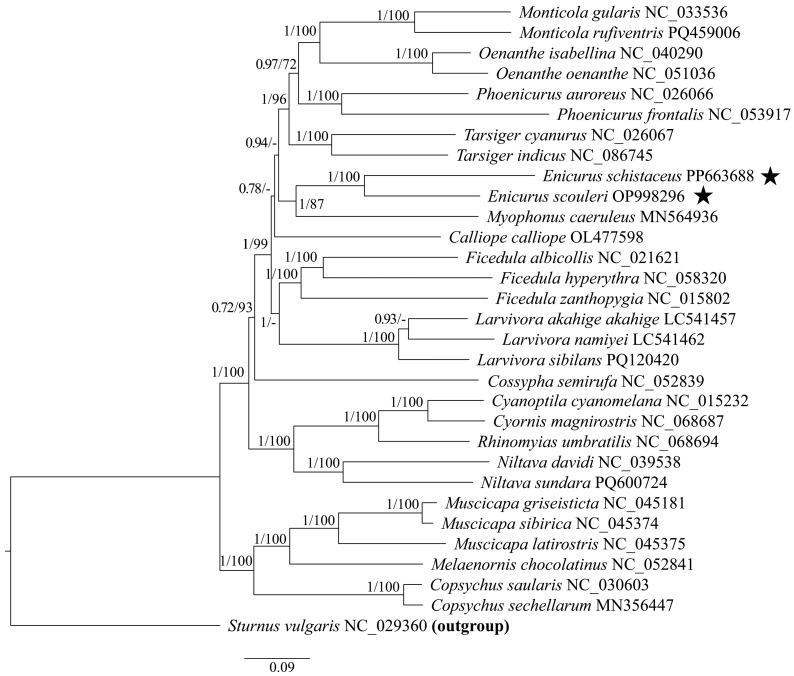
Mitochondrial phylogeny of 30 Muscicapidae species. The newly described species in this work are marked with an asterisk next to their names. Node support: Bayesian PP/ML BS; “-” indicates the values of maximum likelihood bootstrap proportions < 70.

The RSCU-based clustering analysis revealed that the 30 Muscicapidae species were partitioned into two major clades (Figure 8). The first clade comprised six species, including two *Enicurus* species, one *Cossypha* species (*Cossypha semirufa*), and three *Larvivora* species. Within this clade, *E. scouleri* and *E. schistaceus* formed a strongly supported sister pair that diverged near the root, with *Larvivora* and *Cossypha* constituting a distinct subgroup indicating shared codon usage patterns among the six species. The second clade encompassed the remaining 24 Muscicapidae species, including a distinct subgroup comprising two *Ficedula* species (*Ficedula albicollis* and *Ficedula hyperythra*), *Phoenicurus auroreus*, and *Oenanthe isabellina*. The other 20 species, belonging to 14 genera (*Muscicapa*, *Melaenornis*, *Ficedula*, *Monticola*, *Tarsiger*, *Calliope*, *Oenanthe*, *Copsychus*, *Phoenicurus*, *Niltava*, *Cyanoptila*, *Cyornis*, *Rhinomyias*, and *Myophonus*), belonged to another group. The phylogenetic trees were constructed using the mitochondrial protein-coding genes (mtPCGs) to validate the findings. As depicted in Figure 9, although the phylogenetic tree was similarly divided into two major branches, there were several discrepancies between the topological structures of the two diagrams, particularly when comparisons were made at the genus level. Three *Muscicapa* species, two *Copsychus* species, and one *Melaenornis* species (*Melaenornis chocolatinus*) formed the first branch, showing a close phylogenetic relationship. The five species were classified into different clades of RSCU-based clustering lineage, especially the three species of *Muscicapa*. Three genera—*Monticola*, *Oenanthe*, and *Phoenicurus*—had close evolutionary relationships and exhibited distant relationships within the RSCU-based clustering lineage. *E. scouleri* and *E. schistaceus* clustered with *Myophonus caeruleus*, indicating their close evolutionary relationship. Examination of closely related species, including *Cyanoptila cyanomelana*, *Cyornis magnirostris*, and *Rhinomyias umbratilis*, revealed convergent codon usage patterns, reinforcing the principle that evolutionary proximity predicts codon bias similarity. However, *Calliope calliope* exhibited discordant placement between RSCU-based clustering and sequence-based phylogeny, with the latter more accurately reflecting the established taxonomic relationships among the 30 Muscicapidae species. The discrepancies suggested that while CUB generally tracks phylogeny, localised genomic mutations at sequence loci contribute substantially to evolutionary divergence.

## 4. Discussion

The evolution of species and genes has led to CUB, which varies across genomes, genes, and even within individual genes. It often differs substantially between the nuclear and organellar genomes of the same species [57,58]. Considering that mitochondrial genomes evolved under distinct yet related evolutionary forces [59], we assessed CUB in *E. scouleri* and *E. schistaceus*. Our findings indicated a strong A/C preference for both nucleotide composition and codon usage in *Enicurus* mitochondrial genomes. Therefore, we speculated that codons in the mitochondrial genome of *Enicurus* species are more biased towards ending with A and C. Similar results were observed in fish (*O. punctatus* and *O. fasciatus*), avian (superfamily Certhioidea), and two *Tarsiger* species (*Tarsiger indicus* and *Tarsiger cyanurus*) [27,29,60]. However, in other animal and plant taxa, such as vertebrates (reptiles and hynobiids), invertebrates (*Japanagallia* species, *Rhingia* species, *Krisna* species, *B*. *mori*, and *Cyphochilus crataceus*), and plants (*Hemerocallis citrina*, and *Medicago* species), mitochondrial genome codons are more biased towards ending with A and T [14,15,20,24,28,30,61,62,63]. This demonstrated that codon usage patterns vary to some extent across different species while mitochondrial genome CUB is similar among the phylogenetically closely related species [57].

The convergence of high-frequency and optimal codons at the A/C terminations further substantiated the strong third-position compositional bias characteristic of *Enicurus* mitochondrial genomes. The four shared optimal codons (AUC, GUA, UCC, and CGA) suggested that the genetic features of *Enicurus* species were highly conserved and consistent with their close phylogenetic relationship. CUB regulates translational accuracy and efficiency via optimal codon selection, thereby affecting gene expression [64]. Therefore, identifying the optimal codons can improve mitochondrial gene transformation and protein expression efficiency [65,66], providing a theoretical basis and practical guidance for subsequent research on *Enicurus* genetics, development, and species conservation.

Nucleotide usage patterns vary across species and genes. Concordantly, the two *Enicurus* species exhibited divergence in multiple codon usage indices, including CAI, CBI, FOP, ENC, and GC3s, indicating that the synonymous CUB varied intraspecifically. In addition, codon base composition was significantly correlated with CBI, CAI, FOP, GC3s, Aromo, and Gravy, indicating its influence on codon bias. For example, strong GCall-GC3 correlations in both species (*E. scouleri*: *r* = 0.73; *E. schistaceus*: *r* = 0.81) implied genome-wide compositional constraints in shaping codon usage. However, *E. schistaceus*-specific associations between CAI (implying translational efficiency) and GCall/Aromo (*r* = 0.73, 0.67) suggested enhanced selective optimisation for translation in this species. Conversely, the universal negative correlations between hydrophobicity (Gravy) and codon bias indices (CBI and FOP) indicated that highly hydrophobic proteins maintain weaker codon optimisation, possibly due to reduced translational demand or structural constraints. The ENC statistic measures the degree of CUB, ranging from 20 (extreme bias) to 61 (no bias). Conventionally, ENC ≤ 35 is interpreted as strong codon preference in a genome or gene [47,67,68]. In this study, the CAI, CBI, and FOP values were all significantly below 0.5, and the average ENC values were greater than 35 (36.19 and 36.99, respectively). The codon usage indices collectively indicated a weak CUB in the mitochondrial genomes of *Enicurus* species.

CUB has been extensively studied, with natural selection and mutation pressure recognised as the two primary driving forces, the former being predominant in eukaryotes [3,4,5,6,69,70,71,72,73]. Our multi-method analysis, encompassing neutrality plots, ENC plots, PR2 plots, and COA, converged on a unified conclusion that natural selection dominates *Enicurus* mitochondrial codon usage evolution, consistent with studies on other vertebrates, including birds, reptiles, and amphibians [28,29,30]. Given the high energy demands of birds for flight and migration, the synthesis rate of respiratory chain proteins in the mitochondria is critical. Preferential use of specific optimal codons can accelerate protein production and confer survival advantages in various environments. Future research should elucidate the synergistic interplay between natural selection and mutation pressure in shaping codon bias across avian mitochondrial genomes, thereby comprehensively revealing the forces driving mitochondrial genome evolution and their consequences in avian adaptation and survival.

Specialised in energy metabolism and evolving at moderate rates, the mitochondrial genome is an information-rich molecular marker for phylogenetic reconstruction. The comparative analysis of CUB across organisms facilitates species classification and molecular evolution research, revealing that codon bias is correlated with phylogenetic relationship [74,75]. Concatenated mitochondrial protein-coding sequences resolved the Muscicapidae phylogeny with high confidence, as evidenced by robust posterior probabilities and bootstrap values across major nodes. Clustering of *E. scouleri*, *E. schistaceus*, and *M*. *caeruleus* strongly supported their close evolutionary affinity, corroborating the findings of Lan et al. (2024) [60]. RSCU-based cluster analysis revealed congruent codon usage patterns among three related species (*C*. *cyanomelana*, *C*. *magnirostris*, and *R*. *umbratilis*), reinforcing the link between CUB and phylogenetic relationships [75]. However, we found topological discrepancies between RSCU clustering and sequence-based phylogenies; for example, *C*. *calliope* occupied divergent positions between the cluster and phylogenetic analyses. One possibility is that due to the rapid radiation experienced by *Calliope* species [76], allelic combinations from diverse genetic backgrounds may modify the transcription and translation efficiency of mitochondrial genes, thereby influencing their codon usage patterns. Notably, the two *Enicurus* species grouped with the three *Larvivora* species and *C*. *semirufa* and shared similar codon usage patterns. *Enicurus* and *Larvivora* (Saxicolinae and Muscicapinae) from Asia and the African forest robin lineage (Erithacinae) containing *C. semirufa* exhibit pronounced terrestrial habits, favoring understory ground and shrub layers [42]. Despite having diverged in the middle to late Miocene (~10–15 Mya) and undergone prolonged independent evolution [76], their transcontinental codon usage convergence implies that similar ecological niches may have exerted comparable selective pressures on metabolic adaptation, leading to convergent mitochondrial codon usage. A comparable pattern has also been documented in the investigation of CUB in three *Medicago* mitochondrial genomes [63]. Here, we hypothesize that the analogous migratory behaviors, open-habitat adaptations, and energy metabolism requirements of these avian species may impose similar selective pressures at the mitochondrial genomic level, thereby driving convergent evolution in codon usage [40,77].

These incongruences between cluster analysis and phylogenetic analysis likely stemmed from fundamental methodological differences, as follows:

(1) RSCU analysis disregards non-biased codon information [15];
(2) RSCU reflects translational selection and not neutral evolution [14,78,79];
(3) RSCU clustering captures synonymous codon frequency patterns that reflect translational optimisation, whereas Bayesian inference relies on nucleotide sequence similarities and explicit evolutionary models;
(4) Codon bias could evolve through lineage-specific pathways that traverse phylogenetic boundaries [80].

Although methodological differences result in topological inconsistencies, the complementary nature of these approaches enriches our understanding of the evolutionary dynamics of codon usage. Therefore, RSCU-based clustering is a valuable adjunct to traditional phylogenetic methods [81,82], with cluster-derived relationships providing critical insights into the mitochondrial genome evolution and codon usage pattern formation in *Enicurus*.

In summary, the current study established natural selection as the dominant evolutionary force governing CUB in two *Enicurus* mitochondria, laying the groundwork for family-wide investigations of codon usage and phylogeny. Priority should be given to comprehensive taxon sampling in future studies to resolve the taxonomic status of problematic Muscicapidae genera.

## 5. Conclusions

This study comprehensively analysed the CUB in the mitochondrial genomes of two *Enicurus* species to explore their usage patterns and evolutionary determinants. It revealed a weak but structured CUB in *Enicurus* mitochondria, marked by an A/C-ending preference and low-expression signatures. Multi-method analyses (ENC plot, neutrality plot, and PR2 plot) converged on natural selection as the dominant evolutionary force, with mutational pressure as a significant modifier. Species-specific optimal codon sets (16 versus 11) with four shared A/C-rich codons could offer practical tools for biological applications. Integration of sequence phylogeny and RSCU clustering revealed nuanced CUB-evolution dynamics, enhancing our understanding of mitochondrial genome evolution and its implications in Muscicapidae systematics, ecological adaptation, and conservation strategies.

## Figures and Tables

**Table 1:** The codon usage parameters of ten mitochondrial genes in two *Enicurus* species.

Species	Base Composition at the Third Position of the Synonymous Codon (%)				GC Content (%)			
	T3s	C3s	A3s	G3s	GC1	GC2	GC3	GCall
*E. scouleri*	25.29	40.35	39.29	13.34	47.02	46.35	46.51	46.63
*E. schistaceus*	34.57	34.26	33.32	18.41	42.46	47.45	44.80	44.90

**Table 2:** Frequency distribution of ENC_ratio_.

Class Range	Class Mid Value	*E. scouleri*		*E. schistaceus*	
		Class Number	Frequency	Class Number	Frequency
0.20–0.25	0.225	0	0	1	0.1
0.26–0.30	0.275	0	0	0	0
0.31–0.35	0.325	2	0.2	3	0.3
0.36–0.40	0.375	3	0.3	2	0.2
0.41–0.45	0.425	4	0.4	4	0.4
0.46–0.50	0.475	1	0.1	0	0
Total		10	1	10	1

**Table 3:** RSCU values of ten mitochondrial genes codons in two *Enicurus* species.

Amino Acid	Codon	*E. scouleri*				*E. schistaceus*			
		RSCU	RSCU-H	RSCU-L	ΔRSCU	RSCU	RSCU-H	RSCU-L	ΔRSCU
Ala	GCU	0.7568	0.3478	1.0612	−0.7134	0.6897	0.7	0.8485	−0.1485
	GCC	1.9459	2.6087	1.7959	**0.8128**	1.8544	1.8	2.303	−0.503
	GCA	1.1892	1.0435	1.1429	−0.0994	1.3333	1.5	0.8485	**0.6515**
	GCG	0.1081	0	0	0	0.1226	0	0	0
Cys	UGU	0.6429	2	0	**2**	0.3571	0	0	0
	UGC	1.3571	0	2	−2	1.6429	2	2	0
Asp	GAU	0.549	0.5	0.4615	0.0385	0.72	0	0.7692	−0.7692
	GAC	1.451	1.5	1.5385	−0.0385	1.28	2	1.2308	**0.7692**
Glu	GAA	1.7662	1.8	1.6667	**0.1333**	1.7436	1.6364	1.8824	−0.246
	GAG	0.2338	0.2	0.3333	−0.1333	0.2564	0.3636	0.1176	**0.246**
Phe	UUC	1.6988	1.4444	1.6364	−0.192	0.3855	0.5217	0.5882	−0.0665
	UUU	0.3012	0.5556	0.3636	**0.192**	1.6145	1.4783	1.4118	0.0665
Gly	GGU	0.4734	0.6154	0.2	**0.4154**	0.5087	0	0.9412	−0.9412
	GGC	0.9467	0.9231	0.6	**0.3231**	0.9711	0.2353	0.9412	−0.7059
	GGA	1.8225	2.4615	2.6	−0.1385	1.9422	3.2941	2.1176	**1.1765**
	GGG	0.7574	0	0.6	−0.6	0.578	0.4706	0	**0.4706**
His	CAU	0.2078	0	0.2353	−0.2353	0.4533	0.6667	0.5455	**0.1212**
	CAC	1.7922	2	1.7647	**0.2353**	1.5467	1.3333	1.4545	−0.1212
Ile	AUU	0.449	0.32	0.4651	−0.1451	0.607	0.3902	0.8	−0.4098
	AUC	1.551	1.68	1.5349	**0.1451**	1.393	1.6098	1.2	**0.4098**
Lys	AAA	1.9459	2	1.8889	**0.1111**	1.8919	1.8571	2	−0.1429
	AAG	0.0541	0	0.1111	−0.1111	0.1081	0.1429	0	**0.1429**
Leu	UUA	0.4218	0.2069	0.875	−0.6681	0.704	0.7333	0.5684	**0.1649**
	UUG	0.1371	0	0	0	0.1051	0.0667	0	0.0667
	CUU	0.5589	0.3448	0.75	−0.4052	0.5779	0.5333	0.7579	−0.2246
	CUC	1.2337	1.1724	1.25	−0.0776	1.0508	1.2667	0.8211	**0.4456**
	CUA	3.3849	4	2.9375	**1.0625**	3.268	3.1333	3.7895	−0.6562
	CUG	0.2636	0.2759	0.1875	**0.0884**	0.2942	0.2667	0.0632	**0.2035**
Met	AUA	1.5349	0.9333	1.5172	−0.5839	1.5285	1.7143	1.0588	**0.6555**
	AUG	0.4651	1.0667	0.4828	**0.5839**	0.4715	0.2857	0.9412	−0.6555
Asn	AAU	0.1622	0	0.1053	−0.1053	0.5766	0.8571	0.5333	**0.3238**
	AAC	1.8378	2	1.8947	**0.1053**	1.4234	1.1429	1.4667	−0.3238
Pro	CCU	0.5111	0	1.125	−1.125	0.7458	0.6154	0.5926	0.0228
	CCG	0	0	0	0	1.0621	0.9231	1.037	−0.1139
	CCA	2	2.2609	1.75	**0.5109**	2.1243	2	2.3704	−0.3704
	CCC	1.4889	1.7391	1.125	**0.6141**	0.0678	0.4615	0	**0.4615**
Gln	CAA	1.8372	2	1.8947	**0.1053**	1.8353	1.875	1.8667	0.0083
	CAG	0.1628	0	0.1053	−0.1053	0.1647	0.125	0.1333	−0.0083
Arg	CGU	0.4	0	0.4444	−0.4444	0.4	0	0.3636	−0.3636
	CGC	0.9333	1.1429	1.3333	−0.1904	0.8	0	1.4545	−1.4545
	CGA	2.3333	2.8571	1.7778	**1.0793**	2.4667	4	1.8182	**2.1818**
	CGG	0.3333	0	0.4444	−0.4444	0.3333	0	0.3636	−0.3636
Ser	UCU	0.5902	0.2308	0.5	−0.2692	0.7531	0.6486	0.4865	**0.1621**
	UCC	1.9918	2.3077	2.125	**0.1827**	1.7322	1.9459	1.6216	**0.3243**
	UCA	2.041	1.8462	2.25	−0.4038	2.159	2.2703	2.2703	0
	UCG	0.123	0.2308	0	**0.2308**	0.1004	0	0	0
	AGU	0.2213	0	0.375	−0.375	0.251	0.1622	0.3243	−0.1621
	AGC	1.0328	1.3846	0.75	**0.6346**	1.0042	0.973	1.2973	−0.3243
Thr	ACU	0.6667	0.4706	1.0667	−0.5961	0.8182	1.3103	0.5556	**0.7547**
	ACC	1.8222	1.8824	1.8	**0.0824**	1.7576	1.5172	1.5556	−0.0384
	ACA	1.4667	1.6471	1.0667	**0.5804**	1.3788	1.1034	1.7778	−0.6744
	ACG	0.0444	0	0.0667	−0.0667	0.0455	0.069	0.1111	−0.0421
Val	GUU	0.5676	0	0.2963	−0.2963	0.8219	0.4444	0.4615	−0.0171
	GUC	1.5135	1.6667	2.0741	−0.4074	1.0959	1.3333	1.2308	**0.1025**
	GUA	1.4865	2.3333	1.4815	**0.8518**	1.6712	2.2222	2	**0.2222**
	GUG	0.4324	0	0.1481	−0.1481	0.411	0	0.3077	−0.3077
Trp	UGA	1.8333	1.7778	1.7333	0.0445	1.9524	2	2	0
	UGG	0.1667	0.2222	0.2667	−0.0445	0.0476	0	0	0
Tyr	UAU	0.5055	0.8	0.5714	**0.2286**	0.383	0	0.5455	−0.5455
	UAC	1.4945	1.2	1.4286	−0.2286	1.617	2	1.4545	**0.5455**

Note: underlined numbers indicate RSCU > 1, bolded numbers indicate ΔRSCU > 0.08.

**Table 4:** Optimal codons of ten mitochondrial genes in two *Enicurus* species.

Amino Acid	Codon	*E. scouleri*			*E. schistaceus*		
		Number	RSCU	ΔRSCU	Number	RSCU	ΔRSCU
Leu	CUA ***	321	3.3849	1.0625			
Leu	CUC **				100	1.0508	0.4456
Ile	AUC *	190	1.5510	0.1451	179	1.3930	0.4098
Met	AUA ***				94	1.5285	0.6555
Val	GUC *				40	1.0959	0.1025
Val	GUA ***	55	1.4865	0.8518	61	1.6712	0.2222
Ser	UCC *	81	1.9918	0.1827	69	1.7322	0.3243
Ser	AGC ***	42	1.0328	0.6346			
Pro	CCA ***	90	2.0000	0.5109			
Pro	CCC ***	67	1.4889	0.6141			
Thr	ACC *	123	1.8222	0.0824			
Thr	ACA ***	99	1.4667	0.5804			
Ala	GCA ***				87	1.3333	0.6515
Ala	GCC ***	126	1.9459	0.8128			
Tyr	UAC ***				76	1.6170	0.5455
His	CAC *	69	1.7922	0.2353			
Gln	CAA *	79	1.8372	0.1053			
Asn	AAC *	102	1.8378	0.1053			
Lys	AAA *	72	1.9459	0.1111			
Asp	GAC ***				32	1.2800	0.7692
Glu	GAA *	68	1.7662	0.1333			
Arg	CGA ***	35	2.3333	1.0793	37	2.4667	2.1818
Gly	GGA ***				84	1.9422	1.1765

Note: * indicates ΔRSCU > 0.08, and ** indicates ΔRSCU > 0.3, and *** indicates ΔRSCU > 0.5.

## Data Availability

The datasets generated for this study can be found in GenBank with the following accession numbers: OP998296 and PP663688. The datasets analysed during the current study are available on the NCBI GenBank database (https://www.ncbi.nlm.nih.gov/genbank/, accessed on 20 March 2025).

## Supplementary Materials

The following supporting information can be downloaded at: https://www.mdpi.com/article/10.3390/genes17050518/s1, Supplementary File S1: Table S1. GenBank accession numbers and species information for 30 *Muscicapidae taxa*. Supplementary File S2: Table S2. Nucleotide substitution models and partitioning schemes identified using PartitionFinder 2.1.1. Supplementary File S3: Table S3. Characteristic codon parameters of mitochondrial coding genes in the two *Enicurus* species.

## Abbreviations

The following abbreviations are used in this manuscript:

GC1, GC2, GC3	GC contents at the first, second, and third codon positions, respectively
GC3s	GC content at synonymous (silent) third codon positions
GC12	Mean GC content of the first and second codon positions
GCall	Overall genomic GC content
CAI	Codon adaptation index
CBI	Codon bias index
CUB	Codon usage bias
ENC	Effective number of codons
GRAVY	Grand average of hydropathicity
Aromo	Aromaticity of protein
Fop	Frequency of optimal codon
L-sym	Length of synonymous codons
L-aa	Length of amino acids
RSCU	Relative synonymous codon usage
ENC_exp_	Expected ENC values
ENC_obs_	Observed ENC values
BI	Bayesian Inference
ML	Maximum Likelihood
