# Supplementary material for: Codon Usage Bias and Phylogenetic Analysis of the Mitochondrial Genomes in Two Enicurus Species
Source: Genes (Basel). 2026 Apr 28;17(5):518. doi: 10.3390/genes17050518 (PMC13205950; doi:10.3390/genes17050518)
Supplement: Supplementary file 1 [file genes-17-00518-s001.zip › Supplementary File S1 Table S1. GenBank accession numbers and species information for 30 Muscicapidae taxa.pdf]

Supplementary File S1: Table S1. GenBank accession numbers and species information for 30 Muscicapidae taxa.

Genus	Species	Genbank Accession Number
Calliope	*Calliope calliope*	OL477598
Copsychus	*Copsychus saularis*	NC_030603
Copsychus	*Copsychus sechellarum*	OL477598
Cossypha	*Cossypha semirufa*	NC_052839
Cyanoptila	*Cyanoptila cyanomelana*	NC_015232
Cyornis	*Cyornis magnirostris*	NC_068687
Enicurus	*Enicurus scouleri*	OP998296
Enicurus	*Enicurus schistaceus*	PP663688
Ficedula	*Ficedula hyperythra*	NC_058320
Ficedula	*Ficedula zanthopygia*	NC_015802
Ficedula	*Ficedula albicollis*	NC_021621
Larvivora	*Larvivora akahige akahige*	LC541457
Larvivora	*Larvivora namiyei*	LC541462
Larvivora	*Larvivora sibilans*	PQ120420
Melaenornis	*Melaenornis chocolatinus*	NC_052841
Monticola	*Monticola gularis*	NC_033536
Monticola	*Monticola rufiventris*	PQ459006
Muscicapa	*Muscicapa griseisticta*	NC_045181
Muscicapa	*Muscicapa latirostris*	NC_045375
Muscicapa	*Muscicapa sibirica*	NC_045374
Myophonus	*Myophonus caeruleus*	MN564936
Niltava	*Niltava davidi*	NC_039538
Niltava	*Niltava sundara*	PQ600724
Oenanthe	*Oenanthe isabellina*	NC_040290
Oenanthe	*Oenanthe oenanthe*	NC_051036
Phoenicurus	*Phoenicurus auroreus*	NC_026066
Phoenicurus	*Phoenicurus frontalis*	NC_053917
Rhinomyias	*Rhinomyias umbratilis*	NC_068694
Tarsiger	*Tarsiger cyanurus*	NC_026067
Tarsiger	*Tarsiger indicus*	NC_086745
