# Supplementary material for: Codon Usage Bias and Phylogenetic Analysis of the Mitochondrial Genomes in Two Enicurus Species
Source: Genes (Basel). 2026 Apr 28;17(5):518. doi: 10.3390/genes17050518 (PMC13205950; doi:10.3390/genes17050518)
Supplement: Supplementary file 1 [file genes-17-00518-s001.zip › Supplementary File S2 Table S2. Nucleotide substitution models and partitioning schemes identified using PartitionFinder 2.1.1..pdf]

Supplementary File S2: Table S2. Nucleotide substitution models and partitioning schemes identified using PartitionFinder 2.1.1.

**Dataset - MrBayes**		
Subset	Partition names	Best Model
1	*ND3*_position1, *ND1*_position1, *ND4L*_position1	GTR+I+G
2	*ATP6*_position2, *ND3*_position2, *ND4*_position2, *ND1*_position2, *ND4L*_position2	TVM+I+G
3	*ND1*_position3, *ND2*_position3	GTR+I+G
4	*ND2*_position1	TVM+I+G
5	*ND2*_position2	GTR+I+G
6	*COX3*_position1, *COX1*_position1	GTR+I+G
7	*COX1*_position2	HKY+I+G
8	*COX1*_position3	GTR+I+G
9	*COX2*_position1, *CYTB*_position1	TVM+I+G
10	*COX2*_position2, *CYTB*_position2, *COX3*_position2	TVM+I+G
11	*COX2*_position3	TVM+I+G
12	*ATP8*_position1	TRN+I
13	*ATP8*_position2, *ND5*_position2	GTR+I+G
14	*ATP8*_position3, *COX3*_position3	GTR+I+G
15	*ND4*_position1, *ATP6*_position1	TVM+I+G
16	*ND3*_position3, *ATP6*_position3	K81UF+I+G
17	*ND4*_position3, *ND4L*_position3, *ND5*_position3	GTR+I+G
18	*ND5*_position1	GTR+I+G
19	*CYTB*_position3	TVM+G
20	*ND6*_position1	GTR+I+G
21	*ND6*_position2	GTR+I+G
22	*ND6*_position3	GTR+G
