# Supplementary material for: Codon Usage Bias and Phylogenetic Analysis of the Mitochondrial Genomes in Two Enicurus Species
Source: Genes (Basel). 2026 Apr 28;17(5):518. doi: 10.3390/genes17050518 (PMC13205950; doi:10.3390/genes17050518)
Supplement: Supplementary file 1 [file genes-17-00518-s001.zip › Supplementary File S3 Table S3. Characteristic codon parameters of mitochondrial coding genes in the two Enicurus species..pdf]

Supplementary File S3: Table S3. Characteristic codon parameters of mitochondrial coding genes in the two *Enicurus* species.

**gene**	**GC1(%)**		**GC2(%)**		**GC3(%)**		**GC3s(%)**		**GC(%)**		**CAI**		**CBI**		**Fop**		**ENC**		**L_sym**		**L_aa**		**Gravy**		**Aromo**	
	**X**	**HB**	**X**	**HB**	**X**	**HB**	**X**	**HB**	**X**	**HB**	**X**	**HB**	**X**	**HB**	**X**	**HB**	**X**	**HB**	**X**	**HB**	**X**	**HB**	**X**	**HB**	**X**	**HB**
ND1	52.76	53.40	41.41	41.36	46.01	40.56	46.20	40.60	46.73	45.11	0.15	0.14	0.06	-0.01	0.44	0.39	38.95	39.42	325	323	325	323	0.79	0.76	0.13	0.13
ND2	45.24	44.38	44.09	44.67	42.36	43.06	42.50	43.10	43.90	44.04	0.14	0.13	0.05	0.03	0.44	0.43	39.07	35.11	346	346	346	346	0.69	0.70	0.09	0.09
COX2	56.58	57.02	37.28	37.72	46.49	40.79	46.70	41.00	46.78	45.18	0.18	0.18	0.09	0.00	0.47	0.42	39.39	39.60	227	227	227	227	0.31	0.31	0.09	0.09
ATP6	57.02	55.26	39.04	38.16	45.18	35.09	45.40	35.20	47.08	42.84	0.10	0.10	-0.03	-0.06	0.36	0.34	33.27	41.96	227	227	227	227	0.99	1.09	0.07	0.07
COX3	55.56	54.96	42.91	42.91	47.51	45.98	47.50	46.00	48.66	47.96	0.17	0.20	0.10	0.11	0.48	0.49	36.57	37.36	261	261	261	261	0.46	0.47	0.18	0.18
ND3	52.99	52.14	40.17	40.17	42.74	31.03	43.10	31.00	45.30	41.14	0.13	0.13	0.00	0.03	0.40	0.41	32.43	30.94	116	116	116	116	0.87	0.87	0.13	0.13
ND4	53.59	51.09	42.48	41.83	45.10	40.09	45.10	40.10	47.06	44.34	0.11	0.12	0.01	0.00	0.41	0.40	35.12	38.60	459	459	459	459	0.77	0.81	0.08	0.08
ND5	45.71	45.87	42.57	41.91	49.17	46.20	49.10	46.30	45.82	44.66	0.16	0.15	0.13	0.10	0.50	0.48	34.36	35.59	605	605	605	605	0.67	0.73	0.10	0.11
CYTB	54.07	52.76	38.58	38.58	48.03	46.72	48.20	46.80	46.89	46.02	0.15	0.16	0.07	0.06	0.45	0.45	34.69	35.31	380	380	380	380	0.68	0.67	0.13	0.13
ND6	57.23	56.07	43.35	42.20	50.29	41.04	50.00	40.70	50.29	46.44	0.16	0.16	-0.12	-0.12	0.31	0.31	38.08	36.08	172	172	172	172	1.29	1.29	0.12	0.12
